# CD44 Targeting Mediated by Polymeric Nanoparticles and Combination of Chlorine TPCS_2a_-PDT and Docetaxel-Chemotherapy for Efficient Killing of Breast Differentiated and Stem Cancer Cells In Vitro

**DOI:** 10.3390/cancers12020278

**Published:** 2020-01-23

**Authors:** Elisa Gaio, Claudia Conte, Diletta Esposito, Elena Reddi, Fabiana Quaglia, Francesca Moret

**Affiliations:** 1 Department of Biology, University of Padova, 35121 Padova, Italy; elisa.gaio@studenti.unipd.it (E.G.); elena.reddi@unipd.it (E.R.); 2 Drug Delivery Laboratory, Department of Pharmacy, University of Napoli Federico II, 80131 Napoli, Italy; claudia.conte@unina.it (C.C.); diletta.esposito@unina.it (D.E.); fabiana.quaglia@unina.it (F.Q.)

**Keywords:** photodynamic therapy, chemotherapy, combination therapy, cancer stem cells, targeted nanoparticles, hyaluronic acid, mammospheres

## Abstract

The presence of rare but highly tumorigenic cancer stem cells (CSCs) within the tumors is recognized as one of the major reasons of failure of conventional chemotherapies, mainly attributed to the development of drug resistance and increasing metastatic potential. Here, we propose a therapeutic strategy based on the simultaneous delivery of docetaxel (DTX) and the photosensitizer meso-tetraphenyl chlorine disulfonate (TPCS_2a_) using hyaluronic acid (HA) coated polymeric nanoparticles (HA-NPs) for the targeting and killing of CD44 over-expressing breast cancer (BC) cells, both differentiated and CSCs (CD44^high^/CD24^low^ population), thus combining chemotherapy and photodynamic therapy (PDT). Using the CD44^high^ MDA-MB-231 and the CD44^low^ MCF-7 cells, we demonstrated the occurrence of CD44-mediated uptake of HA-NPs both in monolayers and mammosphere cultures enriched in CSCs. Cell treatments showed that combination therapy using co-loaded NPs (HA@DTX/TPCS_2a_-NPs) had superior efficacy over monotherapies (HA@DTX-NPs or HA@TPCS_2a_-NPs) in reducing the self-renewal capacity, measured as mammosphere formation efficiency, and in eradicating the CSC population evaluated with aldehyde dehydrogenase activity assay and CD44/CD24 immunostaining. In summary, these in vitro studies demonstrated for the first time the potential of the combination of DTX-chemotherapy and TPCS_2a_-PDT for killing CSCs using properly designed NPs.

## 1. Introduction

The complex physiology of tumor tissues, the coexistence of cell subpopulations with distinct phenotypes, and the development of intrinsic/acquired drug resistance are some of the causes of failure of conventional cancer therapies. In tumors, a large fraction of differentiated cells coexist with a small fraction of cancer stem cells (CSCs) that, similarly to normal stem cells, have the capability of self-renewal and differentiation into phenotypically diverse cancer cells [1]. CSCs are highly tumorigenic and drive cancer initiation, progression, recurrence, and metastasis in a wide panel of tumor types, including breast cancers (BC). In fact, accumulating evidence has led to the concept of BC as a disease of BCSCs since the plasticity of BCSCs plays a pivotal role in determining the evolution and the prognosis of the disease [2]. It is also clear that the concomitant killing of differentiated cells and targeting/killing of CSCs must be the driving goals in developing new therapeutic strategies aiming at assuring long-lasting disease remission and long-term survival of BC patients.

Currently, some clinical protocols are based on the administration of multiple drugs in a single cocktail or in separated doses as well as the combination of different treatment modalities [3]. Among clinically approved cancer therapies, photodynamic therapy (PDT) is a compelling curative treatment for several solid tumors, able to efficiently reduce bulky cancer masses [4]. PDT is based on three intrinsically non-toxic components that exert cytotoxicity only when combined together. These are a photosensitizer (PS), visible light, and molecular oxygen. The PS activation with appropriate wavelengths of light stimulates the production of highly reactive oxygen species (ROS), mainly singlet oxygen (^1^O_2_), that cause localized oxidative stress, cell death, and activation of immunological and inflammatory responses in the treated tissue [5]. In comparison to conventional cancer treatments, PDT is selective because: (i) Owing to limited diffusion in the tissues of the produced ROS, the oxidative damage is restricted to the sites of PS localization in the tumor; (ii) light is delivered exclusively to desired areas using optical fibers; (iii) the PSs localize and are retained mainly in neoplastic lesions, especially if delivered as proteins/peptide-conjugates [6,7] or loaded in properly engineered nanoparticles (NPs) [8]. In recent years, it has been reported that the combination of PDT with chemotherapy produced superior therapeutic efficacy over mono-therapies, in several cancer cell lines in vitro and in in vivo tumor models, including BC [9,10,11,12]. In addition, several studies highlighted that the therapeutic effects of the combined treatment is further improved if the chemotherapeutic and PS are co-encapsulated in nanometric delivery systems [13,14,15]. In line with these observations, we have recently reported the successful synergic killing of docetaxel (DTX)-sensitive and -resistant cancer cells in vitro [16], using biodegradable hyaluronic acid (HA)-decorated double-layered NPs co-loaded with DTX [17] and the PS meso-tetraphenyl chlorine disulfonate (TPCS_2a_) [18]. NPs were conceived in order to: (i) Transport the hydrophobic DTX in the inner poly (D,L-lactide-*co*-glycolide) (PLGA) core; (ii) favor electrostatic association of the amphiphilic PS to a positively charged polyethyleneimine (PEI) layer covering the core; (iii) exploit active targeting, via the HA receptor CD44 over-expressed on cancer cells, covering the NP surface with a layer of HA [19]; (iv) guarantee synergic interaction of the transported drugs through the easy modification of drug ratio inside NPs [20]. In combination therapies, the drug ratio is a crucial factor for obtaining synergism, but in previous work, we were able to synergistically kill CD44-overexpressing cervix cancer HeLa and triple negative BC MDA-MB-231 cells by loading NPs with an optimized DTX:TPCS_2a_ ratio that significantly improved treatment efficacy with respect to combination of chemotherapy and PDT using free drug formulations. The improvement was particularly important with DTX-resistant cells. Based on these results and on evidence that BCSCs eradication is particularly difficult because of slow proliferation rate, low sensitivity to antimitotic agents, and over-expression of different membrane efflux pumps causing resistance to chemotherapy [21], we made the hypothesis that it might be possible to increase the killing of BCSCs with the combination of DTX-chemotherapy and TPCS_2a_-PDT using HA-covered NPs for the targeting of BCSCs in addition to differentiated BC cells. In principle, the antimitotic DTX should be active against the bulky population of fast-proliferating cells while TPCS_2a_-mediated PDT should be equally effective toward fast-proliferating and slow-proliferating BCSCs. Furthermore, the killing of BCSC might be potentiated by CD44 targeting through HA engrafted on NP surface, since BCSC population is given by cells with a high expression of CD44 and low expression of CD24 (CD44^high^/CD24^low^) [22]. Thus, in the present work, we studied for the first time the capability of HA-targeted DTX and TPCS_2a_-loaded NPs (HA@DTX/TPCS_2a_-NPs) to kill proliferating BC cells, cultured as monolayers, as well as CSC-enriched tridimensional BC spheres (e.g., mammospheres) [23] obtained from MCF-7 and triple negative MDA-MB-231 BC cells. The results showed that our HA-targeted DTX/TPCS_2a_ nanoformulation is more effective than monotherapies in: (i) killing proliferating BC cells; (ii) reducing their stemness and self-renewal capacity; (iii) reducing the total numbers of BCSCs.

## 2. Results

### 2.1. NP Preparation and Characterization

In preliminary experiments we observed that MCF-7 cells are less sensitive to treatment with free DTX (IC_50_ 0.134 μg/mL) compared to MDA-MB-231 cells (IC_50_ 0.016 μg/mL) (Tables S1 and S2, and [16]). In addition, it is well known that in tridimensional cell cultures and aggregates (e.g., mammospheres) the drug accumulation inside the cells is generally reduced in comparison to monolayers [24]. On these premises, the 1:35 DTX/TPCS_2a_ ratio inside NPs, that had been optimized for MDA-MB-231 cells, was modified by keeping un-altered the loading of TPCS_2a_ and increasing that of DTX. Using the method already described in [16], we were able to prepare NPs containing DTX/TPCS_2a_ with the 1:5 ratio. The properties of the new nanoformulation are reported in Table 1 and compared with those of NPs loaded with a single drug (HA@DTX-NPs and HA@TPCS_2a_-NPs) used as controls.

All the formulations showed drug entrapment efficiencies higher than 95%, sizes comprised in the 160–210 nm range, a polydispersity index (PI) of 0.2 and a high negative zeta potential, due to the presence of the negatively charged HA external layer. The release of the drugs from NPs was checked in cell culture medium enriched with 10% serum in order to reproduce the experimental conditions of in vitro cell studies. As demonstrated for HA@DTX/TPCS_2a_-NPs with a drug ratio 1:35 [16], in this novel formulation the release of the PS was slow, thus highlighting its strong association with NP. Concerning the chemotherapeutic, the 70% of entrapped DTX was released in 72 h, thus showing a fast burst effect in the first 6 h of incubation (Figure S1).

### 2.2. CD44-Mediated Endocytosis Is Involved in the Cellular Uptake of NPs and Transported Drugs

**Figure 1:**
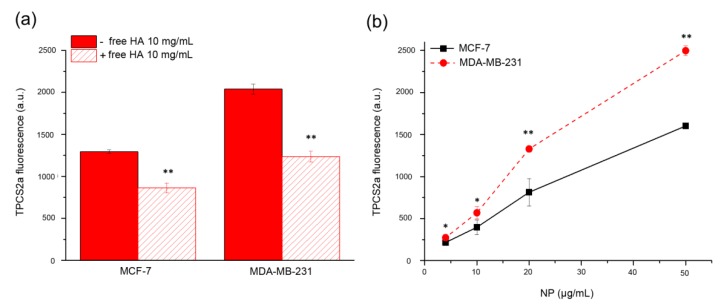
CD44-mediated endocytosis of HA@TPCS_2a_-NPs. (**a**) Uptake of HA@TPCS_2a_-NPs (50 μg/mL NPs) in MDA-MB-231 and MCF-7 cells after 2 h of incubation at 37 °C in medium with or without 10 mg/mL of free HA. (**b**) Concentration-dependent uptake of HA@TPCS_2a_-NPs in MDA-MB-231 and MCF-7 cells after 24 h of incubation at 37 °C. Data are expressed as means ± SD of at least three independent experiments, carried out in triplicate; * *p* < 0.05, ** *p* < 0.001 (Student’s t test).

To assess the effective involvement of HA in enhancing specific drug uptake in CD44 over-expressing cells, we first incubated monolayers of MDA-MB-231 (high CD44 expression) and MCF-7 (low CD44 expression) cells for 2 h with 50 μg/mL of HA@TPCS_2a_-NPs. As shown in Figure 1a, the mean fluorescence signal of TPCS_2a_ was significantly higher in CD44 over-expressing MDA-MB-231 cells with respect to MCF-7. Furthermore, in the competition experiment carried out with cells exposed to an excess of free HA, to saturate CD44 receptors before NP addition, we estimated that at least one third of the PS was effectively taken up by receptor-mediated endocytosis of NPs. Thus, HA-NPs can be used as carriers to improve drug uptake in CD44^+^ cells with an efficiency related to the expression level of the receptor. The increase of the incubation time to 24 h and the NP dose did not affect the higher PS accumulation in MDA-MB-231 cells with respect to MCF-7 cells (Figure 1b).

### 2.3. Combination Therapy Using HA@DTX/TPCS_2a_-NPs Is Effective Toward Differentiated MCF-7 Cells

**Figure 2:**
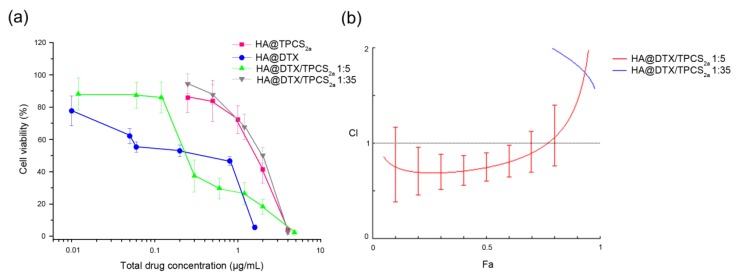
Cytotoxicity of single and combined treatments in differentiated MCF-7 cells cultured as monolayers. (**a**) Dose-response curves of cells incubated for 24 h with single drugs or their combination loaded in HA-NPs and irradiated with 1 J/cm^2^ of red light (600–800 nm) when PDT was part of the treatment. After additional 24 h in drug-free medium, cell viability was measured with the MTS assay. Total drug concentration is referred to DTX + TPCS_2a_ concentration. Data are expressed as mean percentage ± SD of at least three independent experiments, carried out in triplicate. (**b**) Plots of combination index (CI) vs. fraction affected (Fa) relative to cells treated with HA@DTX/TPCS_2a_-NPs loaded with DTX and TPCS_2a_ in the 1:35 (blue) or 1:5 (red) molar ratio.

In a previous work [16], we reported that the combination of DTX-chemotherapy and TPCS_2a_-PDT produced synergistic killing of differentiated CD44 over-expressing HeLa and MDA-MB-231 cells. Here we found that the analysis, according to the Chou and Talalay method [25], of the viability of MCF-7 cells (Figure 2a) treated with the HA-NPs co-loaded with the 1:35 DTX/TPCS_2a_ molar ratio revealed antagonism (Figure 2b, blue line, CI > 1: antagonism) instead of synergism. The antagonistic interaction, very likely correlates with: (i) lower sensitivity to DTX of MCF-7 in comparison to the previously used cell lines and consequently the need to increase the loading of DTX inside NPs, and (ii) scarce NP internalization because of low contribution of CD44-mediated uptake, with respect to MDA-MB-231 cells. These hypotheses are supported by the data of Figure 2b showing that when the DTX/TPCS_2a_ ratio was increased to 1:5, synergism between PDT and chemotherapy was observed (red curve, CI < 1: synergism) also in MCF-7 cells.

As visible in the drug-response curves in Figure 2a and Figure S2, and considering the Dm (or IC_50_) values of Table S1, the killing efficiency of PDT alone using HA@TPCS_2a_-NPs was significantly reduced with respect to that of free TPCS_2a_ as a consequence of the reduced PS uptake (Figure S3).

### 2.4. Combination Therapy Using HA@DTX/TPCS_2a_-NPs Is Effective in Reducing Stemness Capacity and Cancer Stem Cell Population in Mammospheres

**Figure 3:**
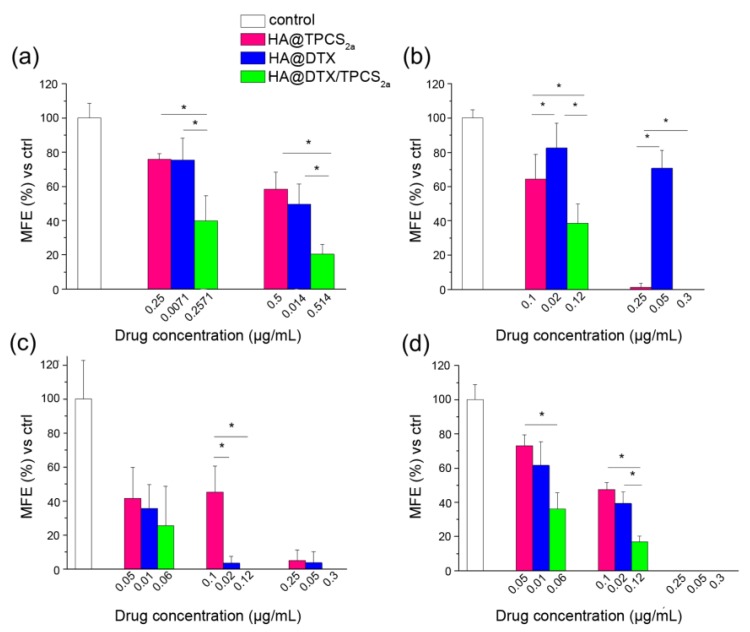
Mammosphere formation efficiency (MFE) after HA-NPs treatments. Percentage MFE measured in first generation mammospheres generated from MDA-MB-231 (**a**) and MCF-7 (**c**) monolayered cells incubated for 24 h with HA@DTX-NPs, HA@TPCS_2a_-NPs and HA@DTX/TPCS_2a_-NPs, irradiated with 1 J/cm^2^, and re-seeded in non-adherent conditions to allow formation of spheres (protocol 1). Percentage MFE measured in second generation mammospheres generated from first generation mammospheres of MDA-MB-231 (**b**) and MCF-7 (**d**) exposed to drug-loaded NPs for 24 h, irradiated with 1 J/cm^2^, and re-seeded in non-adherent conditions (protocol 2). MFE was evaluated after 7 and 4 days from re-seeding for MCF-7 and MDA-MB-231, respectively. Data are expressed as mean ± S.D. of at least two independent experiments, carried out in triplicate; * *p* < 0.05 (one-way ANOVA, Bonferroni’s correction).

MCF-7 and MDA-MB-231 cells were selected because of their different expression profile of the common BCSC markers CD44 and CD24, when cultured as monolayers or tridimensional mammospheres. In fact, while in adherent monolayered MCF-7 cells the CD44^high^/CD24^low^ population (e.g., BCSC population) represents only 1.7%, in MDA-MB-231 almost 93% of cells show BCSC-like phenotype (Figure S4, left). The CD44^high^/CD24^low^ population significantly increased in the mammospheres of first generation (about 6%) formed from MCF-7 cells and lined up at 29% of the cells in the mammospheres of second generation (Figure S4, right). Differently, almost 100% of MDA-MB-231 cells derived from mammospheres were CD44^high^/CD24^low^. The mammosphere formation efficiency (MFE) was used as an indicator of stem cell activity and self-renewal capacity after single treatments, PDT or chemotherapy, or combination of the two using HA-NPs [26]. As explained in detail in Section 4 (Materials and Methods), we measured MFE of first generation mammospheres generated from cells cultured and treated in monolayers (protocol 1), as well as MFE of second generation mammospheres generated from cells of the first generation which had been treated with PDT or chemotherapy or their combination (protocol 2), delivering drugs exclusively with HA-NPs. It is worth to note that for the combined treatment with HA@DTX/TPCS_2a_-NPs of monolayered cells (protocol 1), we selected the drug ratio that produced the best synergism in the individual cell line, namely 1:35 for MDA-MB-231 [16] and 1:5 for MCF-7, while for the treatment of first generation of mammospheres of both cell lines (protocol 2), the 1:5 drug ratio was used as indicated by preliminary experiments. As shown in Figure 3a, the MFE of MDA-MB-231 cells from monolayers exposed to combination therapy using HA@DTX/TPCS_2a_-NPs was significantly lower than that of the cells exposed to PDT or chemotherapy alone, indicating higher efficiency in reducing the numbers of formed spheres. On the contrary, no clear differences were observed in MCF-7 line between DTX alone and combined treatments (Figure 3c). Of note, MDA-MB-231 stemness potential was significantly higher if compared to that of MCF-7 cells treated with comparable drug doses. As an example, while MCF-7 cells treated with PDT following incubation with 0.25 μg/mL TPCS_2a_-loaded in HA-NPs lost almost completely their capacity of re-propagating as spheres, MDA-MB-231 cells treated with the same dose of PDT exhibited only a slight reduction of MFE (about 20%) with respect to untreated cells.

**Figure 4:**
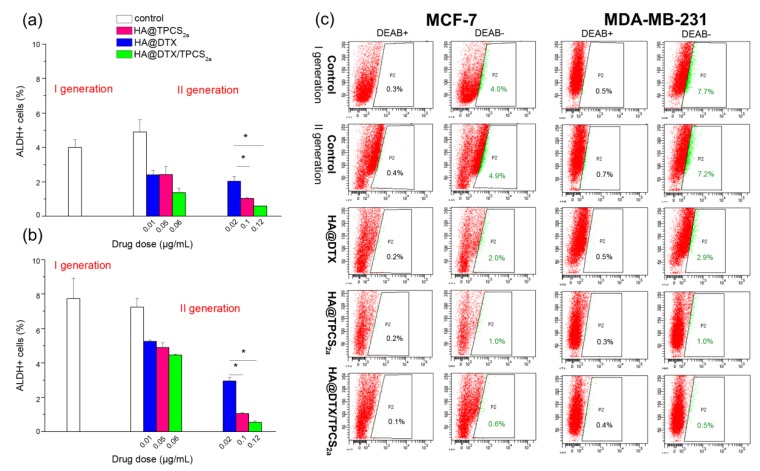
ALDEFLUOR assay in MCF-7 (**a**) and MDA-MB-231 (**b**) cells. Percentages of ALDH positive cells (**a**,**b**) and exemplificative flow cytometry plots (**c**) measured in first and second-generation mammospheres treated with drugs in HA-NPs. First generation mammospheres were incubated for 24 h with HA-NPs, irradiated with 1 J/cm^2^ of red light and re-seeded in non-adherent conditions to allow formation of II-generation spheres. Seven (MCF-7) or 4 (MDA-MB-231) days after the reseed, the population of ALDH^high^ cells was evaluated by gating the ALDH^high^ cells, (green population and percentages) whose fluorescence in flow cytometry plots (DEAB−) exceeded that of the negative controls (DEAB+) stained with the ALDH inhibitor DEAB to control background fluorescence. Flow cytometry plots are referred to mammospheres treated with drug doses of 0.02 μg/mL DTX, 0.1 μg/mL TPCS_2a_, 0.12 μg/mL DTX + TPCS_2a_. Data are expressed as mean ± S.D. of at least two independent experiments; * *p* < 0.05 (one-way ANOVA, Bonferroni’s correction).

Quite different MFEs were measured when the treatments were applied to first generation mammospheres (Figure 3b,d), as exemplified also in the images of MDA-MB-231 and MCF-7 mammospheres treated with equivalent drug concentrations (Figure S5). Indeed, with both MDA-MB-231 (Figure 3b) and MCF-7 (Figure 3d) cells a significantly lower efficiency to form second generation mammospheres was measured using HA@DTX/TPCS_2a_-NPs with respect to single therapies. Moreover, while in MCF-7 we were able to completely abolish sphere formation even with single therapies, in MDA-MB-231 only treatment with HA@TPCS_2a_-NPs or HA@DTX/TPCS_2a_-NPs almost completely abolished the ability to form spheres. To confirm that the significant reduction of forming spheres capacity after treatments correlated with an effective reduction of the CSC populations within treated mammospheres, we measured also ALDH activity, a marker to identify BCSCs [27]. As shown in the summary graphs of Figure 4, BCSC population slightly increased from first to second generation in untreated MCF-7 mammospheres (4% and 4.9%) while it was the same in MDA-MB-231 cells (7.7% and 7.2%). In any case, BCSC population was significantly reduced after HA-NP treatments in both cell lines, even if a different extent of CSC reduction was measured. In MDA-MB-231 cells treated with the highest drug doses considered (e.g., 0.02 μg/mL DTX, 0.1 μg/mL TPCS_2a_), we were able to reduce CSCs by about 2.5, 7, and 14-folds using HA@DTX-NPs, HA@TPCS_2a_-NPs and HA@DTX/TPCS_2a_-NPs, respectively, with respect to untreated second generation mammospheres. Similarly, the same doses of drugs delivered in HA-NPs, determined CSC reduction in MCF-7 by about 2.5, 5, and 8-times. In agreement with the results on the MFE (Figure 3), the less effective treatment was DTX-chemotherapy alone, while its combination with PDT was the best option against BCSCs, since in both cell lines we were able to almost completely eradicate this small but relevant cell population.

The results on ALDH activity in MCF-7 cells agreed with the results on the BCSC population obtained by CD44 and CD24 immunostaining and flow cytometry analyses (Figure S6). Measuring the BCSC population in the second generation mammospheres by this method led to higher percentages than those measured by the ALDEFLUOR assay, but even so MCF-7 showed a 4.5, 5.5, and 6-folds reduction of CSC percentage, with respect to untreated spheres, after treatments with HA@DTX-NPs, HA@TPCS_2a_-NPs, HA@DTX/TPCS_2a_-NPs, respectively, at the DTX dose of 0.02 μg/mL and the TPCS_2a_ dose of 0.1 μg/mL. The treatments with these drug doses decreased the percentages of CSC from 28.6%, of untreated second generation mammospheres, to values of 6.6%, 5.05%, and 4.6%, after incubation with HA@DTX-NPs, HA@TPCS_2a_-NPs and HA@DTX/TPCS_2a_-NPs, respectively, that are similar or slightly lower than the percentage measured in the untreated first generation mammospheres (5.73%).

### 2.5. CD44 Targeting Capability of HA@NPs in Mammospheres

**Figure 5:**
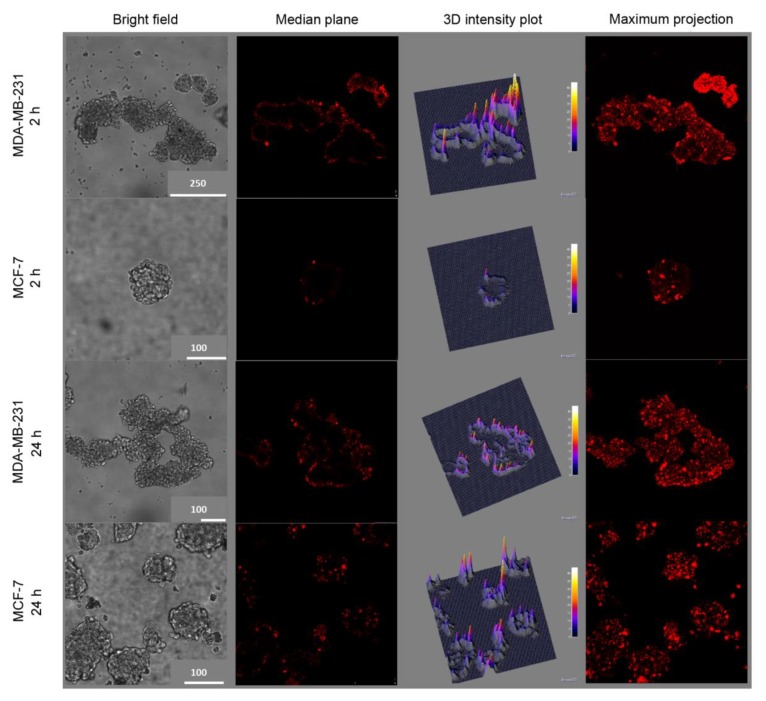
Uptake of HA@TPCS_2a_-NPs in MDA-MB-231 and MCF-7 first generation mammospheres incubated for 2 or 24 h with 50 μg/mL HA@TPCS_2a_-NPs (TPCS_2a_ dose 1.25 μg/mL). Column 1: Bright field images; Column 2: TPCS_2a_ fluorescence at the equatorial plane of mammospheres; Column 3: Three-dimensional reconstruction of TPCS_2a_ fluorescence distribution and intensity in the equatorial plane of the mammospheres; Column 4: Maximum projection obtained from the superimposition of 20 different acquired focal planes. Scale bar unit in the images is µm.

The data reported in Figure 4 indicate that the reduction of BCSC population is slightly more pronounced in MDA-MB-231 cells with respect to MCF-7, especially following treatments with HA@TPCS_2a_-NPs and HA@DTX/TPCS_2a_-NPs. We made the hypothesis that this stronger reduction could be correlated with an increased NP uptake mediated by HA in CD44 over-expressing MDA-MB-231 mammospheres with respect to MCF-7. Thus, we incubated first generation mammospheres with 50 μg/mL HA@TPCS_2a_-NPs (TPCS_2a_ dose 1.25 μg/mL) for 2 or 24 h and then evaluated the TPCS_2a_ fluorescence by confocal microscopy. As visible in the confocal images acquired in the median plane as well as maximum projections shown in Figure 5, the uptake of NPs after 2 h of incubation was significantly higher in MDA-MB-231 with respect to MCF-7 mammospheres, as indicated by the bright red fluorescence signal of TPCS_2a_.

As confirmed also in the fluorescence intensity 3D plot reconstructions, the PS uptake was significantly increased in CD44-overexpressing mammospheres, even if mostly confined to the more external cell layers. Notably, when the incubation time was prolonged up to 24 h (Figure 5), TPCS_2a_ uptake significantly increased also in MCF-7 mammospheres, very likely due to the prevailing of non-specific endocytosis, with respect to HA-mediated uptake of NPs, prolonging the time.

## 3. Discussion

Breast cancer is one of the major causes of death worldwide for women, especially in the case of triple negative BC (TNBC), characterized by poor prognosis and survival due to ineffectiveness of hormonal therapies. Moreover, the aggressive phenotype of this type of cancer is mainly attributed to drug-resistance phenomena and the presence of BCSCs, responsible for the high rate of metastasis formations and tumor relapse after treatments. While the origin and role of BCSC is not completely understood [2], evidence suggests that CSCs derived from the transformation of normal stem cells are responsible for tumor’s aggressiveness higher than that of CSC derived from progenitor cells. Different therapeutic approaches are being developed and proposed, to precisely target and eradicate this small but difficult-to-kill cell population. We have previously demonstrated that TPCS_2a_ delivered through HA-functionalized polymeric NPs exerts markedly higher photosensitizing activity in TNBCs [16]. Interestingly, when TPCS_2a_-PDT was combined to DTX-chemotherapy, by encapsulating the two drugs at optimal ratio within the same NP, a high synergism was found, and TNBC MDA-MB-231 cells were efficiently killed with DTX and TPCS_2a_ doses, respectively, 11- and 6-fold lower than those used in monotherapies for obtaining comparable effects. Nevertheless, very few examples are reported on the use of DTX nanoformulations for the eradication of differentiated cancer and CSCs [28,29,30]. To the best of our knowledge, this is the first time that DTX-chemotherapy is proposed in combination with PDT to fulfill this scope.

The increase of internalized drugs due to CD44 targeting by the HA coating of NPs coupled with the concomitant slow release inside the cells were identified as key factors for the increased performances of the nanoformulation with respect to free drugs. Since many BC cells and BCSCs are characterized by high expression of CD44 while others show low expression, we decided to select MDA-MB-231 as CD44^+^ and adenocarcinoma MCF-7 as CD44^−^ cells, respectively (Figure S4), to study receptor-mediated endocytosis of NPs. As shown in Figure 1, the different level of CD44 expression in these two cell lines correlated with the different extent of cellular uptake of HA-NPs, indicating a clear contribution of HA targeting and CD44-mediated delivery, leading to increased PS internalization in MDA-MB-231 with respect to MCF-7 cells (Figure 1b). This is well supported by the competition experiments (Figure 1a) demonstrating that cell pre-incubation with an excess of free HA to saturate CD44 receptors inhibited TPCS_2a_ uptake to higher extent in MDA-MB-231 compared to MCF-7 as expected based on the expression level of the receptor. Similarly, Gao and co-workers reported the CD44-mediated uptake of the PS chlorin e6 loaded in HA nanocomplexes in CD44^+^ HT-29 cells in vitro and in HT-29 tumor bearing mice [31]. The targeting capability of HA-NPs was observed also in mammospheres (Figure 5), a 3D in vitro culture model enriched of BCSCs. In line with the fact that in the first generation of mammospheres about 97% of MDA-MB-231 cells were CD44^high^/CD24^low^ while only approximately 6% of MCF-7 showed this expression profile (Figure S2), after 2 h incubation, the uptake of HA@TPCS_2a_ in MDA-MB-231 was significantly higher than in MCF-7 cells (Figure 5). As found with other tridimensional in vitro models and other PSs [14,32], TPCS_2a_ localized mainly in the more external cells layers of the mammospheres, and penetrated deeper at increasing incubation time. Weak penetration into the core of MCF-7 mammospheres was observed also with coumarin-loaded HA hybrid NPs [33].

To study the efficacy of the combination of DTX-chemotherapy and TPCS_2a_-PDT in reducing cell stemness and to effectively eradicate BCSCs, we used the mammosphere-forming assay, as a convenient in vitro approach to estimate BC cell potential to behave like “stem cells” or ”tumor initiating” cells (TICs) [34]. Toward this aim, we have determined MFE of cells that had been treated as monolayers (protocol 1) or as mammospheres of first generation (protocol 2). As protocol 1 is concerned, we have preliminarily determined the impact of chemotherapy/PDT combination in CD44^low^ MCF-7 cells cultured as monolayers. Differently from monolayers of MDA-MB-231 cells previously studied [16], HA-NPs gave synergistic killing of MCF-7 exclusively if DTX loading inside NPs was increased (Figure 2b), thus modifying DTX:TPCS_2a_ ratio from 1:35 to 1:5. It is worth to note, that the increased drug ratio did not affect NP stability and drug entrapment efficiency (>95% DTX and TPCS_2a_, see Table 1). When we treated cells cultured as monolayer and then re-propagated them as mammospheres, we observed a clear decrease of the MFEs in both cell lines and, especially in MDA-MB-231, the reduction with combined with respect to single treatments was remarkable (Figure 3a,c). In fact, an MFE higher than 50% was maintained when MDA-MB-231 cells were exposed to monotherapies while the same drugs co-delivered through HA-NPs lowered MFE to less than 50%. Generally, the MFE measured in MCF-7 was significantly lower than that observed in MDA-MB-231, but lower was also the impact of combination therapy on MFE reduction. It can be speculated that a correlation occurs between the cytotoxicity induced in monolayered cells and the reduction of sphere-forming capacity. Interestingly, analyzing cell viability and MFE values in the two cell lines we observed that, while in MCF-7 sub lethal doses (e.g., 0.05 μg/mL TPCS_2a_, 0.01 μg/mL DTX; cell viability around 90–80%) of drugs decreased MFE to less than 40%, in MDA-MB-231 cells the same extent of reduction of sphere-forming capacity was observed with drug doses close to IC_50_ (Table S2 and [16]) (e.g., 0.5 μg/mL TPCS_2a_, 0.014 μg/mL DTX). The higher stemness potential demonstrated by survived MDA-MB-231 cells very likely correlates with the intrinsic high metastatic potential of triple negative cells, while the adenocarcinoma MCF-7 cell line is considered non metastatic [35]. It has been also reported that tumor aggressiveness and metastatic potential are associated with elevated ALDH expression, especially in the case of TNBC [36]. This evidence is confirmed in our models by the higher percentage of ALDH^+^ cells counted in untreated mammospheres of MDA-MB-231 with respect to MCF-7 (Figure 4) and, is in agreement with the observations of Laranjo and co-workers that reported lower ALDH expression in MCF-7 mammospheres with respect to those derived from TNBC HCC1806 cells [37]. It must also be pointed out that the higher IC_50_ for DTX measured in MCF-7 monolayers with respect to MDA-MB-231 (Tables S1 and S2) does not correlate with a higher self-renewal/stemness capacity.

Using protocol 2, we treated first generation mammospheres and measured MFEs in the second generation. Basically, by adopting this protocol we tried to simulate the in vivo tumor environment by reproducing the tridimensional arrangement and the BCSC enrichment. As for the first generation mammospheres obtained from cells treated in monolayer, lower MFE values were measured for MCF-7 with respect to MDA-MB-231 cells, at the same drug doses (Figure 3b,d). The combination therapy was clearly more efficient than each single therapy in MCF-7; this was less evident in MDA-MB-231 mammospheres because of high and low sensitivity of the CSC to PDT and DTX, respectively. The high MFE measured following HA@DTX-NP therapy is very likely in agreement with a more specific activity of taxanes toward non-BCSCs rather than toward BCSCs [28,29,38]. In this context, Zhang and colleagues reported that in xenografts of MDA-MB-231Luc treated with DTX (dose approximately IC_50_), the MFE of isolated cells was even higher than that of cells that had been treated with the vehicle alone, due to an increase of TICs after the treatment [39]. The same DTX-treated cells re-implanted into mice gave rise to tumors whose size was increased by 50% with respect to those derived from cells treated with vehicle only. On the other hand, TPCS_2a_-based PDT seems to target more indistinctly all BC populations. Accordingly, Usacheva and coworkers reported an efficient cytotoxicity of methylene blue (MB) NP-mediated PDT toward proliferating and BCSCs, under normoxic and hypoxic conditions [40]. In addition, in a very recent in vitro study, Abrahamse et al. reported the efficacy of aluminum phthalocyanine (AlPcS_mix_)-based PDT for killing not only the bulk cervical cancer cell population but also the CSC population, isolated from HeLa cultured cells [41]. The reduction of MFE that we observed in second generation mammospheres might correlate with a decrease in the numbers of BCSCs or BCSC-like cells in HA-NP treated first generation mammospheres. Accordingly, ALDEFLUOR assay results (Figure 4) showed a significant correlation with MFE data, since a significant decrease of ALDH^+^ (e.g., BCSCs) cells was observed. Of note, using HA@DTX/TPCS_2a_−NPs we were able to completely eradicate the BCSC population in both cell lines, using the total drug dose of 0.12 μg/mL. Similarly, in MCF-7 cells, BCSC population identified by CD44 and CD24 immunostaining is reduced to the level of untreated first generation after treatments (Figure S6). BCSC population decrease/eradication using HA-NPs loaded with DTX/TPCS_2a_ combination represents the key finding of the present work. In fact, we have demonstrated that DTX-resistance found especially in CSC-enriched MDA-MB-231 mammospheres could be eliminated by a combined PDT treatment. On the other hand, the same combination demonstrated superior efficacy than each single therapy also toward proliferating/differentiated BC cells. Of note, those BC cells that survived to combination therapy possess significantly lower self-renewal, stemness potential and sphere-forming capacity.

## 4. Materials and Methods

### 4.1. Chemicals and Reagents

TPCS_2a_ was provided by PCI Biotech AS (Oslo, Norway). DTX was purchased from LC Laboratories (Woburn, MA, USA). Poly (D,L-lactide-*co*-glycolide) (PLGA) (50:50 Resomer RG 502H inherent viscosity 0.16−0.24 dL/g) was purchased from Boehringer Ingelheim (Ingelheim am Rhein, Germany). PEI (MW = 10–25 kDa branched) and Poloxamer 188 (Pluronic^®^ F68) were purchased from Sigma-Aldrich (St. Louis, MI, USA). Acetonitrile and acetone were purchased from Carlo Erba Reagenti (Milan, Italy). Hyaluronic acid (HA, MW < 10 kDa) was a kind gift of Magaldi Life S.r.l. (Salerno, Italy). Dulbecco’s modified Eagle’s medium (DMEM), DMEM/F12 medium, fetal bovine serum (FBS) and B27 mix were purchased from Life Technologies (Milan, Italy).

### 4.2. Preparation and Characterization of NPs

HA@DTX/TPCS_2a_-NPs co-loaded with DTX and TPCS_2a_ were prepared by a layer-by-layer deposition method as previously described by us [13,16]. Briefly, DTX-NPs were prepared by solvent diffusion of an organic phase (10 mg of PLGA and 50 µg or 7.5 µg of DTX in 2 mL of acetone, for obtaining the DTX/TPCS_2a_ ratio 1:5 and 1:35, respectively) in an aqueous phase (4 mL of water added with Pluronic F68 0.1%). After solvent evaporation, the dispersion was split into four Eppendorf tubes and centrifuged at 5000× *g* for 15 min. NPs in each Eppendorf were coated with 125 μL of a PEI water solution (1 mg/mL), re-centrifuged at 2800× *g* for 15 min, and then 25 µL of a TPCS_2a_ water solution (0.4 mg/mL) was added. Thereafter, NPs were coated with a layer of HA through the addition of 100 µL of a HA water solution (1 mg/mL) in each Eppendorf tube maintaining a constant interval of 15 min between each addition. The NP preparations were freeze-dried for 24 h and the recovery yield of the production process was evaluated on an aliquot of NPs by weighting the solid residue. Results of the quantification are expressed as the ratio of the actual NP weight to the theoretical polymer plus drug weight × 100. Unloaded NPs, HA@DTX-NPs and HA@TPCS_2a_-NPs were prepared for control experiments. NP characterization was carried out as described in ref. [16].

### 4.3. In Vitro Release of TPCS_2a_ and DTX

The release of TPCS_2a_ was followed at 37 °C; 1 mg of NPs was dissolved in 1 mL of DMEM added with 10% FBS. At predetermined time intervals, the sample was centrifuged at 17000× *g* for 20 min. TPCS_2a_ release was assessed in the supernatant by spectrophotometry at wavelength of 414 nm. A TPCS_2a_ calibration curve in the range 0.05–5 μg/mL was constructed in the release medium. The results from three independent experiments are expressed as % release vs. time ± SD. Release of DTX was assessed in 10 mM phosphate buffer saline (PBS) at pH 7.4 containing NaCl (137 mM) and KCl (2.7 mM) by a dialysis method. Four mg of NPs were dispersed in 0.5 mL DMEM + 10% FBS and placed in a dialysis bag Spectra/Por® (MWCO = 3500 Da)(Thermo Fisher Scientific, Waltham, MA, USA). The sample was plunged in 3 mL of PBS (sink condition) and kept at 37 °C up to 72 h. At selected time intervals, 1 mL of release medium was withdrawn and replaced with an equal volume of fresh medium. As control, release profiles of DTX dissolved in EtOH and added of DMEM + 10% FBS were assessed by HPLC as previously reported in [16]. Results are expressed as release % vs. time ± SD of three experiments.

### 4.4. Cell Lines

MCF-7 and MDA-MB-231 human breast cancer cells were purchased from American Type Culture Collection (ATCC, Manassas, VA, USA). The cells were grown in DMEM with Glutamax^TM^ (Life Technologies, Milan, Italy) supplemented with 10% heat inactivated FBS, 100 U/mL streptomycin, and 100 µg/mL penicillin G and maintained at 37 °C under a humidified atmosphere containing 5% CO_2._

### 4.5. Uptake of HA@TPCS_2a_-NPs in Monolayered Cancer Cells

To study the role of CD44 in the uptake of NPs, 5 × 10^4^ cells were grown in 24-well plates for 24 h and incubated for 2 h with 50 μg/mL of NPs. In competition experiments, some samples were pre-incubated for 1 h with 10 mg/mL of free HA in order to saturate the CD44 receptor binding sites before NP addition. At the end of the incubation time, the cells were washed twice with Versene, (Life Technologies, Milan, Italy) detached from the plates with trypsin that was neutralized by the addition of FBS. Cells were centrifuged and resuspended in Versene before measuring TPCS_2a_ fluorescence using a BD FACSCanto^TM^ II flow cytometer (Becton Dickinson, San Jose, CA, USA). A blue laser at 488 nm was used to excite TPCS_2a_ and its fluorescence was detected at wavelengths longer than 670 nm (PerCP channel); for each sample 10^5^ events were acquired and analyzed using the FACSDiva software. The uptake of TPCS_2a_ as a function of NP concentration was measured in cells seeded and handled as described above and incubated for 24 h with increasing concentrations of HA@TPCS_2a_-NPs.

### 4.6. Cytotoxicity and Determination of Combination Index (CI)

Cytotoxicity experiments in MCF-7 cells were performed as described in [16] for MDA-MB-231 cells and the reduction of cell viability was measured with the MTS assay after single or combined treatments. The treatments were carried out with HA@NP-loaded drugs and free drugs for comparison. Briefly, 8000 cells/well were seeded in 96-well plates and, after 24 h of cell growth, the medium was replaced with fresh medium containing increasing concentrations of TPCS_2a_ or DTX or their combination delivered in free form or entrapped in NPs (DTX/TPCS_2a_ ratio 1:35 and 1:5, w/w). Cell viability was measured after 24 h of drug incubation in the dark (time point 24 h) as well as after an additional 24 h in which the cells were kept in drug-free medium (time point 24 + 24 h). For photo-toxicity experiments (PDT), cells were seeded and treated as described above, and at the end of the 24 h of drug incubation, cells were washed twice with PBS Ca^2+^ and Mg^2+^ and irradiated in PBS with a total fluence of 1 J/cm^2^ of red light (600−800 nm) emitted from a Waldmann PDT 1200 lamp (Waldmann Medizintechnik, Villingen-Schwenningen, Germany). The power density was 15 mW/cm^2^ as measured with the radiometer IL 1700 (International Light, Newburyport, MA, USA). Immediately after irradiation, the cells were brought back to the incubator after the replacement of PBS with fresh medium. Cell viability was measured using the MTS test after additional 24 h (phototoxicity; time point 24 + 24 h). Furthermore, in order to assess if the interaction of DTX-chemotherapy and TPCS_2a_-PDT resulted in a synergic effect, CI values were calculated using the CompuSyn software (ComboSyn Inc., Paramus, NJ, USA), based on the Chou and Talalay method [25]. With the experimental data obtained from the cell viability curves, we calculated the fraction affected (Fa) values for each drug concentration tested and analyzed the data on the Compusyn software as already described [16]. For each drug and drug combination, the software calculated also the drug concentration that inhibits cell survival by 50% (IC_50_ or Dm value). 

### 4.7. Generation and Treatment of Mammospheres

MCF-7 and MDA-MB-231 cells were trypsinized from T-75 cm^2^ flasks and then plated in 24-well ultralow attachment flat-bottom plates (Corning, New York, NY, USA) at a density of 5000 and 100,000 viable cells/mL, respectively. Seeding was performed in serum-free DMEM-F12 supplemented with B27 (1:50), 20 ng/mL epidermal growth factor (EGF), 20 ng/mL basic fibroblast growth factor (bFGF) (Peprotech, London, UK), and 5 µg/mL insulin (Sigma Aldrich). MCF-7 and MDA-MB-231 cells were grown for 7 and 4 days, respectively, to form the first generation of mammospheres. To re-propagate the second generation of mammospheres, the first generation mammospheres were collected, gently centrifuged (123× *g*, 10 min) and dissociated into single cells by using 0.25% trypsin/EDTA (Life Technologies). The dissociated cells were re-seeded as indicated for the first generation mammospheres in order to obtain the corresponding second generation. The number of mammospheres formed in each generation was evaluated by counting the number of spheres in the images acquired by bright field microscopy (DMI4000 Leica microscope, Wetzlar, Germany). The mean diameter of each mammosphere was calculated with the LAS AF Lite software, and spheres with a diameter below 100 µm were excluded from counting. For assessing the effects of single and combined therapies using HA-NPs toward BCSCs two different treatment protocols were used. In protocol 1, cells were seeded and treated in monolayer and, immediately after treatment, were re-seeded to form first generation mammospheres. Thus, according to this protocol, cells (5 × 10^5^) were seeded in 60 mm tissue culture dishes and, after 24 h of growth, were treated with DTX or/and TPCS_2a_ delivered in HA-NPs. At the end of the incubation, cells were irradiated in PBS (1 J/cm^2^ of red light) and, immediately after irradiation, were detached from plates and re-seeded in ultra-low attachment plates to allow the formation of the first generation mammospheres. Mammosphere formation efficiency (MFE) was calculated as the number of spheres counted in the first generation, divided by the number of seeded cells, and expressed as percentage means ± SD. In protocol 2, the first generation mammospheres were formed and incubated for 24 h, directly in ultra-low attachment plates, with DTX or/and TPCS_2a_ delivered in HA-NPs. At the end of the incubation time, the spheres were irradiated with 1 J/cm^2^ of red light, when PDT was part of the treatment, and, immediately after irradiation, were dissociated and re-seeded in non-adherent condition to allow the formation of second-generation mammospheres. 

Mammosphere formation efficiency (MFE) was calculated as the number of spheres counted in the second generation, divided by the number of cells seeded, and expressed as percentage (means ± SD).

### 4.8. Analysis of BCSC Population in Mammospheres

CD24 and CD44 immunostaining: First or second generation mammospheres were collected and enzymatically dissociated into single cells; 10^5^ cells were collected, washed twice with cold PBS, and kept in ice. In parallel, the same number of cells was collected from monolayer cells routinely cultured on T-75 cm^2^ flasks. After a second step of washing, a combination of monoclonal antibodies against human CD44 (FITC-conjugated) and CD24 (PE-conjugated) (BD Biosciences, San Diego, CA, USA) was added to the cell suspension and incubated at 4 °C in the dark for 30–40 min following manufacturer’s protocol. Labeled cells were washed with PBS to eliminate unbound antibodies and analyzed by flow cytometry to count the percentage of CD44^high^/CD24^low^ cells.

### 4.9. Aldehyde Dehydrogenase (ALDH) Activity Assay

ALDH enzymatic activity in first- and second-generation mammospheres was measured using AldeFluor Kit (StemCell Technologies, Durham, NC, USA), following the manufacturer’s protocol. Briefly, mammospheres were dissociated into single cells and cells were then re-suspended in Aldefluor Buffer and stained with activated Aldefluor Reagent. As control for background fluorescence, for each sample, 500 µL of cell suspension were transferred in a tube containing diethylaminobenzaldehyde (DEAB), a specific inhibitor of ALDH. After incubation for 40 min at 37 °C, cells were centrifuged at 250× *g* for 5 min, re-suspended in 500 µL of Aldefluor Buffer, and analyzed with the flow cytometer.

### 4.10. Uptake of HA@TPCS_2a_-NPs in Mammospheres

First generation mammospheres were generated as described above and incubated for 2 or 24 h with 1.25 µg/mL of TPCS_2a_ loaded in HA@ NPs (50 µg/mL). The localization of TPCS_2a_ was evaluated by confocal microscopy (Leica SP5) by transferring the mammospheres from 24-well/plates to 35 mm cell imaging dishes (Ibidi, Gräfelfing, Germany) and washing them twice with PBS before visualization. Images were acquired from the top to the bottom of the mammosphere in about 20 different focal planes (z-stack 10 µm). Furthermore, a 3D reconstruction of the distribution of the fluorescence signal in the equatorial plane of the mammospheres was obtained using the software ImageJ and a maximum projection image obtained by superimposition of 20 focal plains using the software LAS AF Lite (Leica).

### 4.11. Statistical Analysis 

The Primer software for biostatistics (McGraw-Hill, Columbus, OH, USA) was used for statistical analysis of the data. The data are expressed as means ± standard deviations (SD) for at least two independent experiments, carried out in triplicate. The difference between two groups of treatments was evaluated by Student’s t test while the differences between more than two groups of treatment was evaluated with one-way ANOVA test with the Bonferroni’s correction and was considered significant for *p* < 0.05.

## 5. Conclusions

In this work, we have reported the successful in vitro elimination of BCSCs using HA-targeted NPs developed for combining DTX-chemotherapy and PDT. HA-mediated uptake of NPs occurred in CD44^high^ cells grown in adherent and 3D conditions thus demonstrating the capability to target also cancer stem-like cells in addition to differentiated cells overexpressing CD44. Interestingly, stemness potential and self-renewal capacity of CSC was strongly hampered when using HA@DTX/TPCS_2a_−NPs. The CSC reduction was significantly lower when cells monolayers or mammospheres were treated with the combination in comparison to chemotherapy or PDT alone. This general trend was confirmed by evaluating the entity of the CSC population by various assays as measure of MFE, ALDH activity, and immunostaining. These results demonstrate that the co-delivery of chemotherapeutics and PSs for PDT using well-designed NPs can potentiate the efficacy of cancer treatments by targeting and killing CSCs and stimulate future studies with HA-nanoformulations especially with in vivo tumor models.

## Figures and Tables

**Table 1:** Properties of NP formulations^a^. HA@DTX/TPCS_2a_-NPs contain DTX and TPCS_2a_ in the molar ratio 1:5; for HA@DTX/TPCS_2a_-NPs loaded with the drugs in the ratio 1:35 refer to our previous work [16].

Formulation	Size ^a^ (nm ± SD)	PI ^b^	ZP ^c^ (mV ± SD)	DTX Actual Loading (μg DTX/mg NPs)	DTX EE ^d^ (%)	TPCS_2a_ Actual Loading (μg TPCS_2a_/mg NPs)	TPCS_2a_ EE ^d^ (%)
HA@DTX	163 ± 5	0.2	−33 ± 2	5.2	98 ± 3	-	-
HA@TPCS_2a_	208 ± 6	0.2	−32 ± 7	-	-	25	97 ± 3
HA@DTX/ TPCS_2a_	205 ± 3	0.2	−37.2 ± 3	4.8	96 ± 4	24	95 ± 3

^a^ Data are expressed as mean ± S.D. of three independent experiments. ^b^ Polydispersity Index. ^c^ Zeta Potential. ^d^ Entrapment Efficiency.

## Supplementary Materials

The following are available online at https://www.mdpi.com/2072-6694/12/2/278/s1, Figure S1: Release of TPCS_2a_ and DTX from HA@DTX/TPCS_2a_-NPs; Figure S2: Cytotoxicity of free drugs delivered in the respective standard solvents in differentiated MCF-7 cells cultured as monolayers; Figure S3: Intracellular uptake of TPCS_2a_ in MCF-7 adherent cells; Figure S4: FACS representative plots of receptor profile (CD44/CD24) expression measured in MCF-7 and MDA-MB-231 cultured in adherent conditions or as mammospheres; Figure S5: Representative bright field images of MCF-7 and MDA-MB-231 second-generation mammospheres derived from first generation mammospheres treated with HA-NPs; Figure S6: CD44/CD24 receptor profiles in MCF-7 mammospheres. Table S1: IC_50_ or Dm value calculated by the Compusyn software in MCF-7 cells exposed to the different DTX and/or TPCS_2a_ formulations. Table S2: IC_50_ or Dm value calculated by the Compusyn software in MDA-MB-231 cells exposed to the different DTX and/or TPCS_2a_ formulations.
