# Supplementary material for: CD44 Targeting Mediated by Polymeric Nanoparticles and Combination of Chlorine TPCS2a-PDT and Docetaxel-Chemotherapy for Efficient Killing of Breast Differentiated and Stem Cancer Cells In Vitro
Source: Cancers (Basel). 2020 Jan 23;12(2):278. doi: 10.3390/cancers12020278 (PMC7072409; doi:10.3390/cancers12020278)
Supplement: Supplementary file 1 [file cancers-12-00278-s001.pdf]

Supplementary Materials

CD44 Targeting Mediated by Polymeric Nanoparticles and Combination of Chlorine TPCS2a-PDT and Docetaxel-Chemotherapy for Efficient Killing of Breast Differentiated and Stem Cancer Cells In Vitro

Elisa Gaio, Claudia Conte, Diletta Esposito, Elena Reddi, Fabiana Quaglia and Francesca Moret

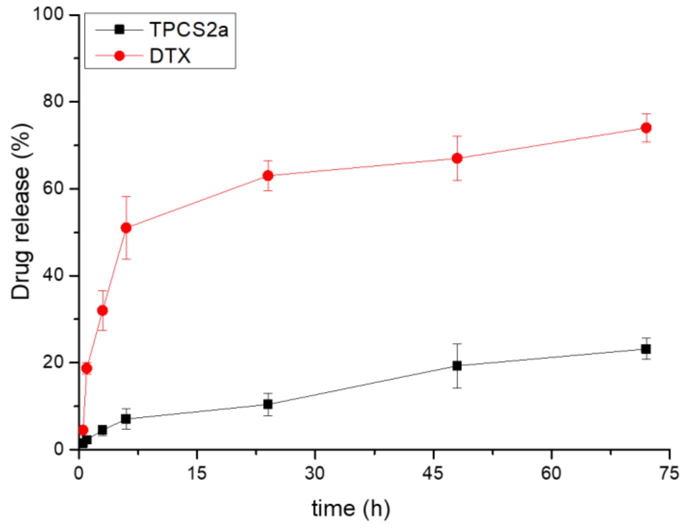

Figure S1. Release of TPCS2a and DTX from HA@DTX/TPCS2a-NPs in DMEM with 10% serum at 37 °C. Data are expressed as mean percentage ± SD of three independent experiments.

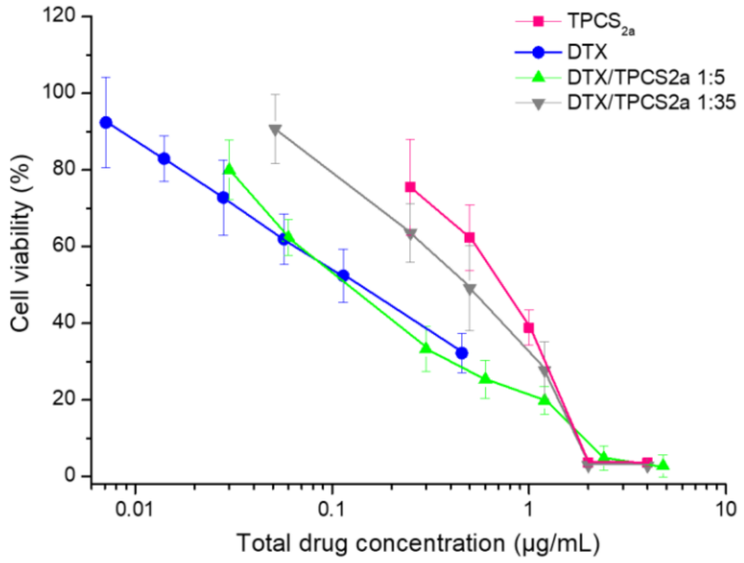

Figure S2. Cytotoxicity of free drugs delivered in the respective solvents in differentiated MCF-7 cells cultured as monolayers. Dose-response curves of cells incubated for 24 h with single drugs or their combination delivered in the free form, irradiated with 1 J/cm² of red light (600–800 nm) when PDT was part of the treatment. After additional 24 h in drug-free medium, cell viability was measured with the MTS assay. Total drug concentration is referred to DTX + TPCS2a concentration. Data are expressed as mean percentage ± SD of at least three independent experiments, carried out in triplicate.

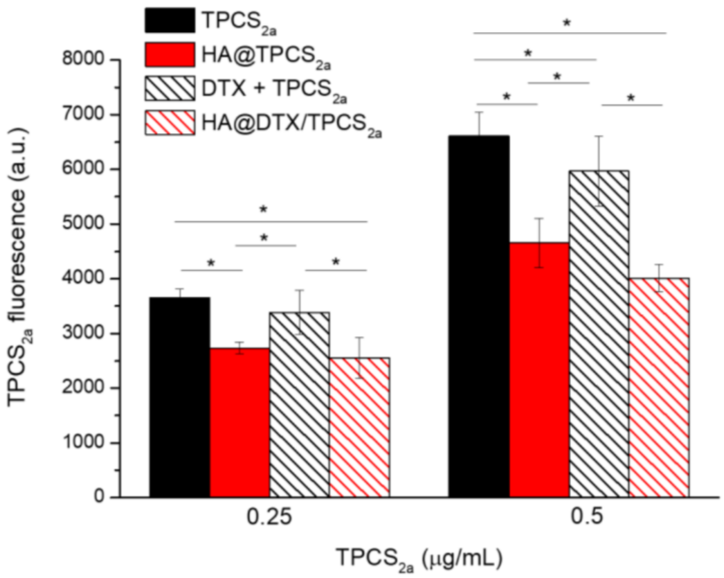

Figure S3. Intracellular uptake of TPCS2a measured in MCF-7 cell monolayers incubated with the photosensitizer delivered in the standard solvent or in HA-NPs, alone or in combination with DTX. The uptake was measured by flow cytometry after 24 h of cell incubation with the drugs. Data are expressed as mean percentage ± SD of at least two independent experiments, carried out in triplicate; * $p < 0.05$ (One-Way ANOVA, Bonferroni's correction).

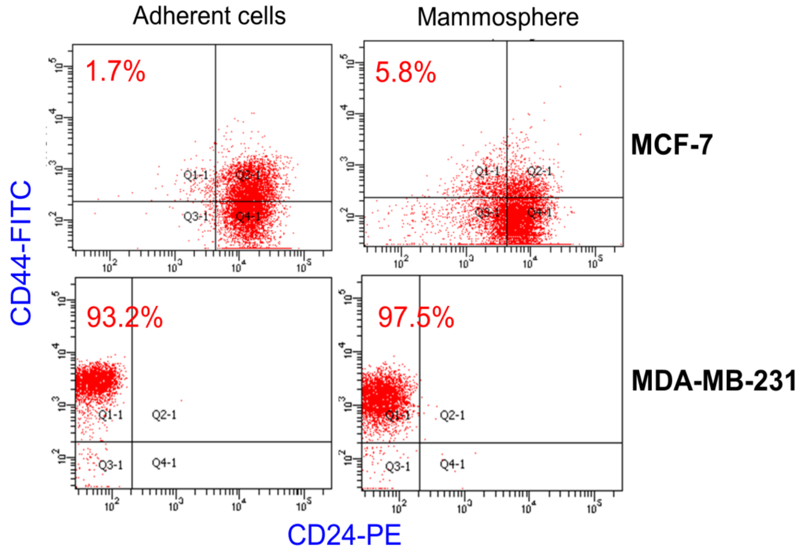

Figure S4. FACS representative plots of receptor profile (CD44/CD24) expression measured in MCF-7 and MDA-MB-231 cultured in adherent conditions or as mammospheres. Cells having a CD44high/CD24low profile, namely cancer stem-like cells, are indicated in Q1-1 with their percentages with respect to the total population indicated by the numbers in red.

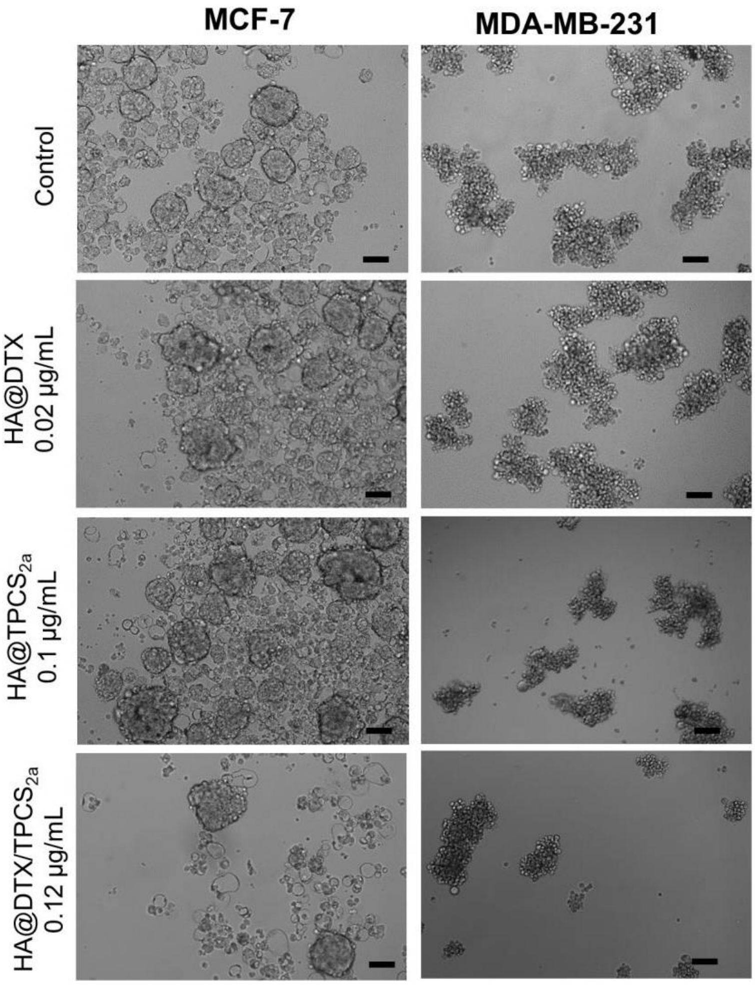

Figure S5. Representative bright field images of MCF-7 and MDA-MB-231 second-generation mammospheres derived from first generation mammospheres treated with HA-NPs. The images were acquired 7 or 4 days after the re-seed for MCF-7 and MDA-MB-231 cells, respectively. Scale bars: 100 µm.

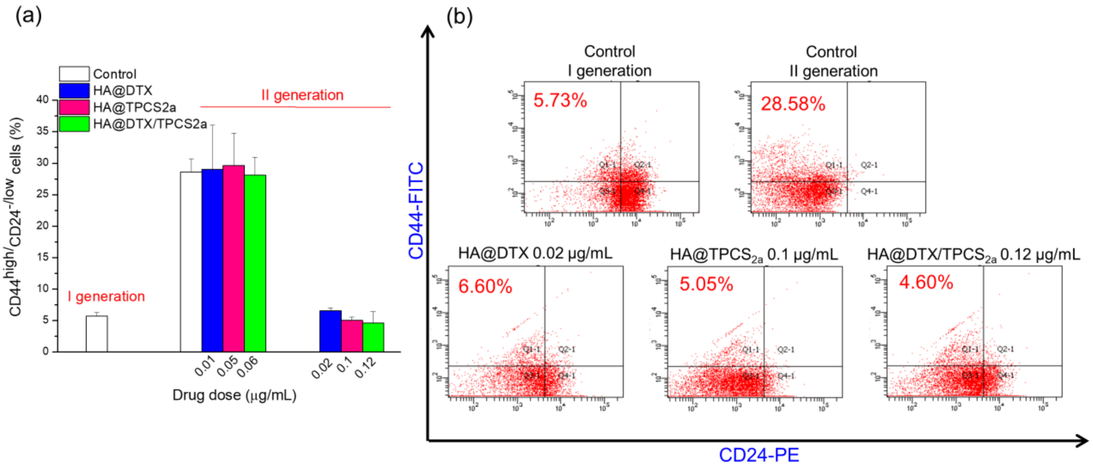

Figure S6. CD44/CD24 receptor profiles in MCF-7 mammospheres. (a) Proportion of CD44high/CD24low cells and (b) representative flow cytometry plots of receptor profile expression measured before (I generation) and after treatment (II generation) with HA@DTX-NPs, HA@TPCS2a-NPs and HA@DTX/TPCS2a-NPs. Mammospheres of the I generation were incubated for 24 h with NPs, irradiated with 1 J/cm2, and re-seeded in non-adherent condition to allow formation of II generation spheres. Before cytometer analysis, cells were stained with a combination of monoclonal antibodies against human CD44 (FITC-conjugated) and CD24 (PE-conjugated). In Q1-1 of the FACS

plots fall those cells having CD44high/CD24low cells receptor expression, namely cancer stem cells. Data are expressed as mean ± S.D. of at least two independent experiments, carried out in triplicate.

Table S1. IC_{50} or Dm value calculated by the Compusyn software in MCF-7 cells exposed to the different DTX and/or $TPCS_{2a}$ formulations. Values indicated drug dose expressed in μg/mL.

Drug Formulation	Total	$TPCS_{2a}$	DTX
$TPCS_{2a}$		0.553	
DTX			0.134
HA@$TPCS_{2a}$		1.108	
HA@DTX			0.144
DTX + $TPCS_{2a}$ (1:35)	0.340	0.330	0.009
DTX + $TPCS_{2a}$ (1:5)	0.137	0.114	0.023
HA@DTX/$TPCS_{2a}$ (1:35)	1.221	1.187	0.034
HA@DTX/$TPCS_{2a}$ (1:5)	0.272	0.226	0.045

Table S2. IC50 or Dm value calculated by the Compusyn software in MDA-MB-231 cells exposed to the different DTX and/or TPCS2a formulations. Values indicated drug dose expressed in μg/mL. These values have been already reported in our previous work ([16] of the main text).

Drug Formulation	Total	$TPCS_{2a}$	DTX
$TPCS_{2a}$		0.332	
DTX			0.016
HA@$TPCS_{2a}$		0.455	
HA@DTX			0.067
DTX + $TPCS_{2a}$ (1:35)	0.200	0.195	0.005
HA@DTX/$TPCS_{2a}$ (1:35)	0.055	0.054	0.001
